# Supplementary material for: MiR-146a in ALS: Contribution to Early Peripheral Nerve Degeneration and Relevance as Disease Biomarker
Source: Int J Mol Sci. 2023 Feb 27;24(5):4610. doi: 10.3390/ijms24054610 (PMC10002507; doi:10.3390/ijms24054610)
Supplement: Supplementary file 1 [file ijms-24-04610-s001.zip › ijms-2201039-supplementary.pdf]

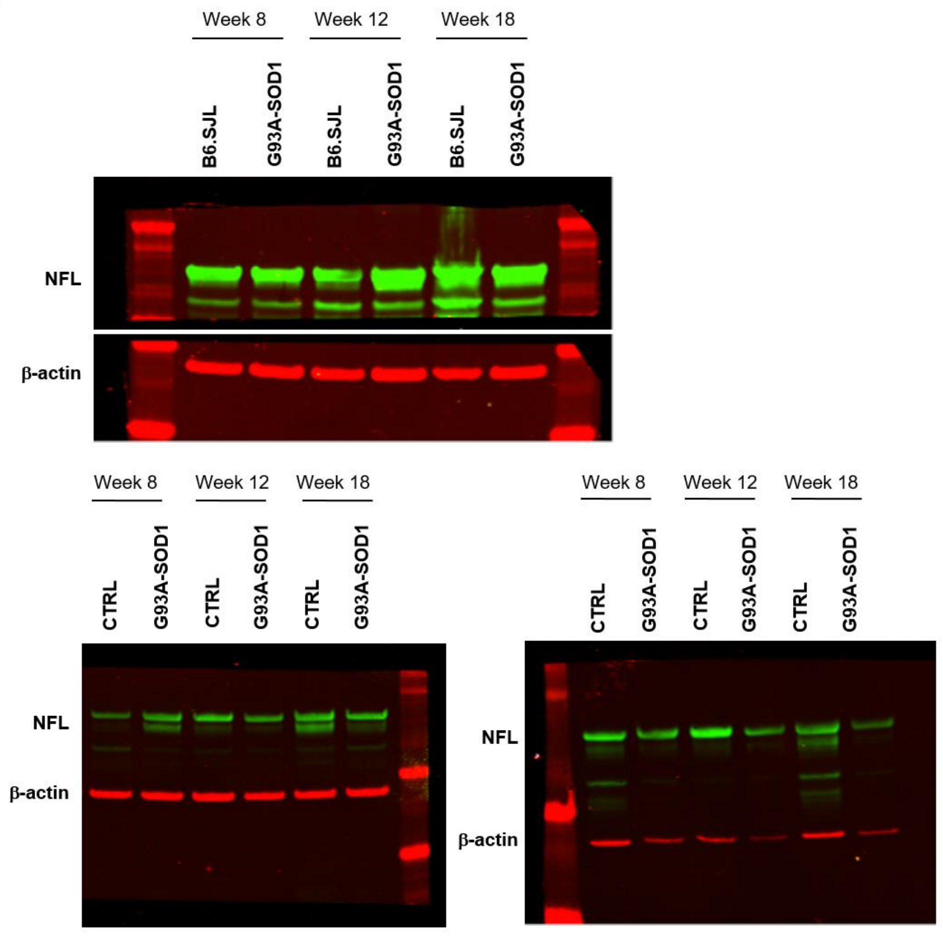

Figure S1. Original images of western blots reported in Figure 3. Representative Western blot analysis of NFL protein in sciatic nerve tissue of G93A-SOD1 and B6.SJL mice at week 8, 12, 18. Western blot images of NFL and β-actin proteins derived from the same electrophoretic gels. Western blot protocol was optimized three times obtaining the same results: one by cutting the membrane into small stripes at proper molecular weight, that were incubated separately with anti-NFL and anti-β-actin antibodies, a "stripe puzzles" method reported in literature [Colciaghi, F. et al. Targeting PSD95-nNOS interaction by Tat-N-dimer peptide during status epilepticus is neuroprotective in MAM-pilocarpine rat model. Neuropharmacology 2019, *153*, 82–97] and two without cutting the membrane.
